# Importance of Bacterial Maintenance Respiration in a Subarctic Estuary: a Proof of Concept from the Field

**DOI:** 10.1007/s00248-018-1244-7

**Published:** 2018-08-22

**Authors:** Kevin Vikström, Johan Wikner

**Affiliations:** 1 Department of Ecology and Environmental Science, Umeå University, SE-901 87 Umeå, Sweden; 2 Umeå Marine Sciences Center, Norrbyn 557, SE-905 71 Hörnefors, Sweden

**Keywords:** Marine, Bacteria, Maintenance, Respiration, Stoichiometry, Model

## Abstract

Bacterial respiration contributes to atmospheric carbon dioxide accumulation and development of hypoxia and is a critical, often overlooked, component of ecosystem function. This study investigates the concept that maintenance respiration is a significant proportion of bacterial respiration at natural nutrient levels in the field, advancing our understanding of bacterial living conditions and energy strategies. Two river-sea transects of respiration and specific growth rates were analyzed representing low- and high-productivity conditions (by in situ bacterial biomass production) in a subarctic estuary, using an established ecophysiological linear model (the Pirt model) estimating maintenance respiration. The Pirt model was applicable to field conditions during high, but not low, bacterial biomass production. However, a quadratic model provided a better fit to observed data, accounting for the maintained respiration at low *μ*. A first estimate of maintenance respiration was 0.58 fmol O_2_ day^−1^ cell^−1^ by the quadratic model. Twenty percent to nearly all of the bacterial respiration was due to maintenance respiration over the observed range of *μ* (0.21–0.002 day^−1^). In the less productive condition, bacterial specific respiration was high and without dependence on *μ*, suggesting enhanced bacterial energy expenditure during starvation. Annual maintenance respiration accounted for 58% of the total bacterioplankton respiration based on *μ* from monitoring data. Phosphorus availability occasionally, but inconsistently, explained some of the remaining variation in bacterial specific respiration. Bacterial maintenance respiration can constitute a large share of pelagic respiration and merit further study to understand bacterial energetics and oxygen dynamics in the aquatic environment.

**Electronic supplementary material:**

The online version of this article (10.1007/s00248-018-1244-7) contains supplementary material, which is available to authorized users.

## Introduction

Bacterial respiration constitutes the largest cause of aquatic oxygen consumption and, therefore, is crucial for the development of oxygen minimum zones and hypoxia worldwide [1–3]. The level and control of bacterial respiration are also crucial for bacterial growth efficiency (BGE), determining the allocation of carbon between bacterial biomass production and CO_2_ emission. Advancement of our understanding of bacterial respiration and its influence on growth efficiency is required to model the global carbon cycle, as well as to counteract hypoxia worldwide and correctly manage the status of the marine environment. In this context, bacterial maintenance respiration has been overlooked.

It has been common in the scientific literature to use a constant (i.e., average) value for BGE between 0.1 and 0.4 when calculating the bacterial carbon demand (cf. [4]). This common practice suggests that respired carbon constitutes a well-defined fraction of assimilated carbon. In contrast, BGE varies at least 50-fold with the level of bacterial production [5–8]. In oligotrophic (e.g., deep ocean) or cold environments (i.e., winter, polar seas) constituting a large and sparsely investigated volume of the ocean, bacterial growth is low and BGE may be below 10% [5, 9–11]. This suggests that maintenance respiration from a global perspective may be substantial.

To understand the level and control of maintenance respiration in the natural environment, we used the best available ecophysiological model for bacterial respiration, the Pirt model [9]. Neijssel and Tempest [12] investigated the influence of nutrient stoichiometry on growth and respiration in *Klebsiella aerogenes* measured in chemostat monocultures. The resulting model describes the relationship between the specific rate of substrate utilization (e.g., O_2_), the specific bacterial growth rate, and the nutrient limitations under energy- and carbon-rich conditions. Maintenance respiration is part of the model and is at significantly higher levels during phosphorus limitation than during nitrogen or carbon limitation. This model was further developed by Pirt [9] to include the influence of a growth-dependent maintenance respiration (*m′*), which suggests a relationship between the rate of substrate utilization (e.g., specific respiration, *R*_sb_) and *μ*, further explained in the “Methods and Materials” section*.* However, to our knowledge, only one study has used the Pirt model and literature values to investigate the control of bacterial growth efficiency, also providing an estimate of bacterial maintenance respiration [13]. Thus, the validity of this postulate in natural aquatic environments remains to be investigated. By simplifying the Pirt model, we observed an opportunity to test its validity in field conditions and to estimate total maintenance respiration for natural bacterial communities. The contribution on a yearly scale was calculated using monitoring data, assuming the marked governance of *R*_sb_ by *μ* in the Pirt model applicable to field conditions.

Current knowledge shows that temperature, nutrient stoichiometry, and the concentration and quality of carbon substrates are the main factors governing bacterial respiration (e.g. [14–18]). A study in the Hudson River suggests that respiration is positively influenced by glucose addition (i.e., carbon substrate) and temperature [8]. Other studies from the Chesapeake Bay and from Canadian lakes show that elevated carbon-to-phosphorus (C:P) ratios increase respiration [19, 20]. Nutrients interacting with temperature also influence bacterial respiration according to earlier studies [21, 22]. Thus, the ratio of the bioavailable C:P and C:N, as well as the quality of carbon substrates, may interact with temperature to influence the level of *R*_sb_ and, thus, the BGE in the field. A general model describing the control of bacterial respiration by these factors is, however, still lacking.

Here, we investigate (1) if the Pirt model can be applied to natural bacterial communities and estimate the bacterial maintenance respiration, (2) the potential contribution from bacterial maintenance respiration over an annual scale using data from a monitoring program, and (3) the influence of nutrient stoichiometry (i.e., C:P) and limitation on the level of bacterial respiration. This was performed by analyzing measurements from subarctic river-sea transects under natural nutrient levels and growth rates of free-living bacteria. By sampling at high- and low-productivity conditions, this provided a large range of bacterial specific growth rates comparable to the annual range. Bacterial maintenance respiration (*R*_m_) was estimated as the *y*-intercept using a simplified Pirt model, assuming bacterial specific growth rates as the main predictor for bacterial respiration.

## Methods and Materials

### Study Site and Sampling

**Fig. 1:**
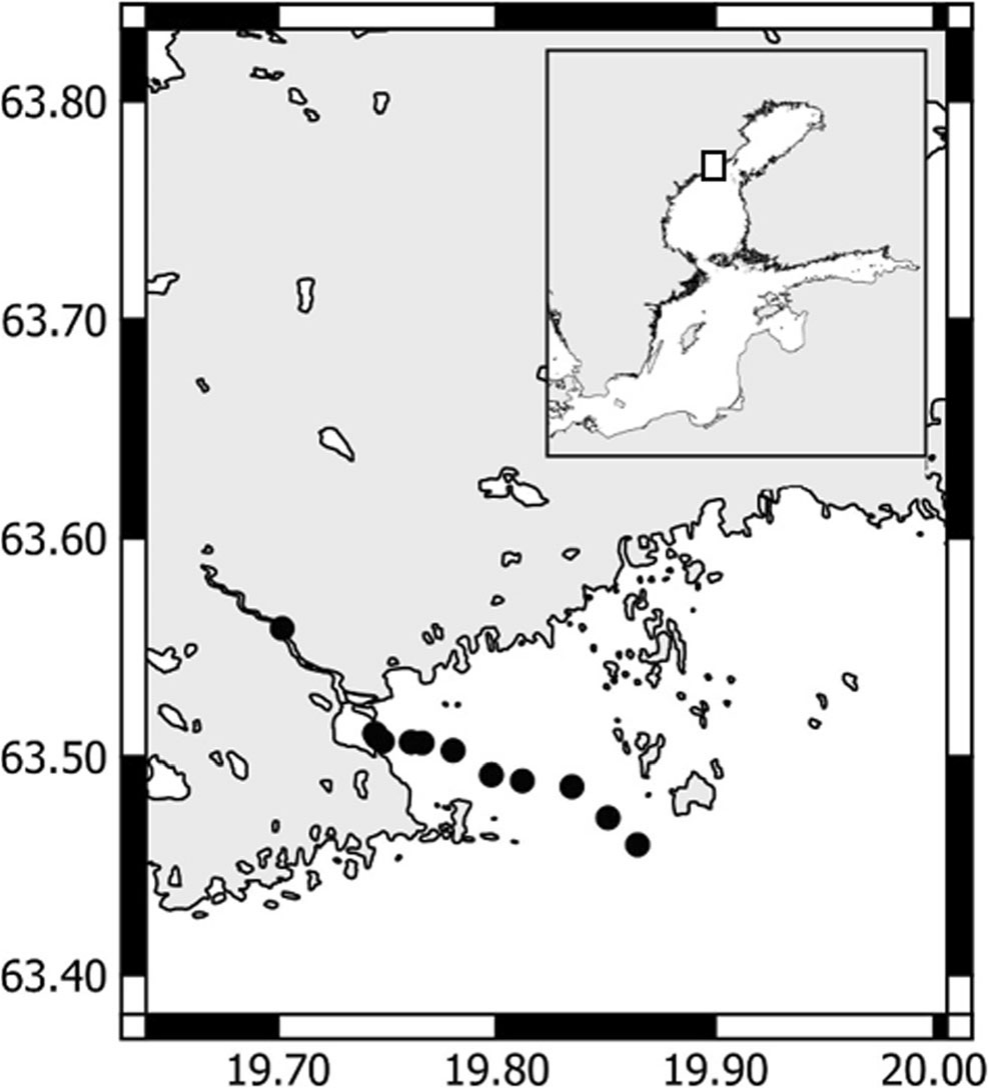
Map of sampling sites (filled circles) in the Öre Estuary, northern Baltic Sea. Decimal degrees for latitude and longitude shown on the axes

Samples were taken from transects with 10 stations at a period of low productivity (April) and 11 stations at a period of high productivity (August) in the Öre Estuary, located in the northwestern Bothnian Sea (a basin in the Baltic Sea, Fig. 1). This subarctic estuary had a high input of dissolved organic carbon (DOC) from the Öre River (12 × 10^6^ [kg total organic carbon] year^−1^ [23]), was generally phosphorus limited [24], and has a mean depth of 10 m and a residence time of 10 days, exchanging water primarily with the main basin of the Bothnian Sea.

**Fig. 2:**
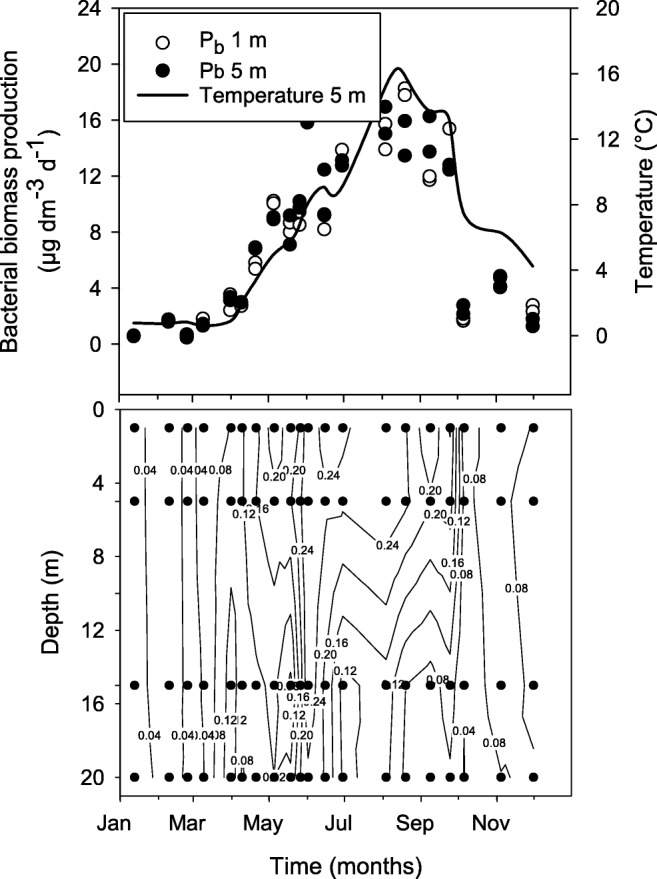
Upper panel: seasonal dynamics of in situ bacterial biomass production and temperature at two surface depths in the Öre Estuary 2015. True duplicate samples from each depth are shown (i.e., parallel sample bottles). Water temperature at 5 m depth is shown as a solid line. Arrows indicate the time of the transect studies of bacterial respiration and growth efficiency. Lower panel: bacterial specific growth rate (*μ*, day^−1^) in the same estuary and year. Filled circles show sampling points

Water samples were obtained from 20 to 22 April 2015, showing poor bacterial growth conditions, and from 3 to 7 August 2015, showing favorable bacterial growth conditions. The productivity level was deduced from the annual bacterioplankton biomass production in situ (Fig. 2, upper panel). Stations were randomized with removal of once chosen stations to avoid time dependency during the sampling week. The experimental design was developed to allow analysis of the whole transect at two depth layers (above and below the pycnocline upper part), maximizing the range of both growth and respiration rates, resulting in total samples of *N* = 20 (April) and *N* = 22 (August), respectively. Differences between individual stations or individual depths could not be compared, as this experimental approach and available resources precluded true replication.

At each station, the pycnocline position, temperature, and salinity were determined with a CTD instrument (conductivity, temperature, and depth; Seabird SBE 19plus, Seabird Electronics, Washington, USA). Water samples (using 5-dm^3^ Niskin bottles) were collected from both the surface and a deeper sample at all stations. Samples were transferred to acid-washed (1 mol dm^−3^ HCl) 4.2-dm^3^ polycarbonate bottles and delivered to the laboratory within 1 h of sampling. The water was filtered using 1.2-μm and 142-mm polycarbonate filters (Isopore, Merck Milllipore™) at − 20 kPa to remove protozoa, phytoplankton, and other larger organisms, enabling the specific measurement of bacterial respiration. All sample bottles and surfaces of the filtering units were soaked in 1 mol dm^−3^ HCl overnight and thoroughly rinsed with Milli-Q water prior to use. The average loss of free-living bacteria due to filtration was 8 ± 11%, estimated as the difference between pre- and post-filtration samples. Variables analyzed from transect sampling were bacterial respiration, bacterial abundance, bacterial production as well as dissolved carbon, nitrogen, and phosphorus.

In addition to the transects, data from an active monitoring program conducted by Umeå Marine Sciences Centre were used to assess the potential annual contribution of maintenance respiration over the full year in the subarctic estuary. Duplicate samples were taken from 1-, 5-, 15-, and 20-m depth with a frequency shown in Fig. 2. The same methods were used for bacterial production (unfiltered) and bacterial abundance (unfiltered) in the monitoring program as in this study. The monitoring data were also used to calculate the coefficient of variation between samples collected from the same depth for chemical variables and bacterial growth and abundance. Data is available from the Swedish Meteorological and Hydrological Institute (https://www.smhi.se/klimatdata/oceanografi/havsmiljodata).

### Estimation of Maintenance Respiration

The Pirt model states a relationship between specific bacterial growth rate (*μ*) and specific bacterial respiration according to Eq. 1.

1 $$ \kern1em {R}_{\mathrm{sb}}=\left(\frac{1}{Y_{\mathrm{g}}}-\frac{m^{\prime }}{\mu_{\mathrm{m}}^{\prime }}\right)\times \mu +{m}_1+{m}^{\prime } $$

The terms within the parentheses represent the respiration-producing energy used for the synthesis of biomass (*R*_bg_), where *Y*_g_ is the maximum growth yield and *μ′*_m_ is the observed maximum growth rate. Maintenance energy is the energy used by the cell to produce basic cell components and structures and is represented by the sum of *m′* and *m*_1_ (hereafter *R*_m_), where *m*_1_ is the constant maintenance energy coefficient, and *m′* is the maintenance energy coefficient representing the growth rate-dependent part of the maintenance energy (all sensu [9]). This equation shows that *m′* increases when *μ* approaches 0, improving the fit of the model to observed data. The physiological meaning of *m′* is not well defined, but Pirt [9] suggests that the formation of energy storage products under energy-sufficient conditions is one explanation. Part of the maintenance energy is consequently described within the parenthesis and is dependent on *μ*, being at its maximum at *μ* = 0. For further derivation of these quantities, please consult the original references cited. Finally, it is important for the interpretation of the results that the sole driver of this model is the specific bacterial growth rate (*μ*). It is, therefore, assumed that *μ* also reflects any influence of taxonomic composition or substrate quality. Bacterial population effects on respiration can therefore not be excluded nor corroborated by this study.

The Pirt model (Eq. 1) was simplified to enable an estimate of the maintenance respiration when *μ* approached 0 for a natural bacterial community and at nutrient levels found in the field according to the following equation:2where *R*_sb_ is the measured cell-specific bacterioplankton respiration, *b* is the expression within the parenthesis in Eq. 1, and *μ* is the measured cell-specific bacterial growth rate. The product *bμ* describes respiration associated with the synthesis of bacterial biomass (i.e., growth or cell division, referred to as *R*_bg_ in Fig. 6). *R*_m_ is the sum of constant (*m*_1_) and growth-dependent maintenance respiration (*m′*), with the latter showing a maximum at *μ* = 0 as explained above. *R*_m_ was estimated as the *y*-axis intercept by model II linear regression with a major axis loss function according to Eq. 2 or the quadratic model (see “Results” section, Eq. 5). Major axis regression takes the uncertainty in both the *x* and *y* terms into account by minimizing the perpendicular distance from the predicted to the observed *y* rather than considering the least squares sum of the squared vertical distances (SPSS^TM^ [25]).

### Calculation of the Annual Influence of Maintenance Respiration

The potential influence of maintenance respiration (*R*_mA_/*R*_sbA_) in the natural environment on the annual scale was calculated as a weighted sum, assuming a distinct dependence on *μ* and using a full annual monitoring data set for observed *μ* (Fig. 2, lower panel) according to Eq. 3.

3 $$ \frac{R_{\mathrm{m}\mathrm{A}}}{R_{\mathrm{sb}\mathrm{A}}}={\sum}_0^{0.28}{f}_{\mathrm{h}}\times \frac{R_{\mathrm{m}}\left(\mu \right)}{R_{\mathrm{sb}}\left(\mu \right)} $$

where *f*_h_ is the fraction of values in *μ* intervals of 0.02 day^−1^ (i.e., calculated from a histogram), encompassing the range of *μ* values observed in the monitoring data [26] from the Öre Estuary ranging from January to December 2015 and the whole water column (i.e., 0.02–0.28 day^−1^, Fig. 2, lower panel). *R*_sb_(*μ*) was calculated with observed *μ* values from the full annual data set, using *R*_m_(*μ*) and *b* as derived from the August data and Eq. 2 (linear model). The same calculation was also done using the nonlinear Eq. 5 (quadratic model, see below) to compare the results of the different models. This calculation assumed that factors other than bacterial specific growth rate had negligible effects on the relationship with *R*_sb_, as postulated by the Pirt model. The calculation also ignored the potentially higher influence of *R*_m_ under low-productivity conditions implied by the April observations.

### Respiration Measurements

Bacterial respiration was measured in one replicate for each sample in acid-washed 1-dm^3^ glass bottles according to Wikner et al. [27] using Aanderaa model 4330 oxygen Optodes™ with a titanium housing rather than the previous plastic-housed model 3835. Controls with autoclaved seawater showed a negligible drift, resulting in a detection limit of 0.9 μmol O_2_ dm^−3^ day^−1^. The detection limit was calculated as the sum of squared standard error (SE) per sample and the squared standard deviation (SD) of the drift, and it fell within the ranges reported elsewhere (e.g., [15, 27–29]). The samples were incubated in the dark at in situ temperature (± 0.05 °C) in a temperature-controlled water bath (Julabo ED, Julabo 13) equipped with an immersion cooler (Julabo FT 200). The oxygen concentration was measured every minute for 12–24 h with continuous stirring using a magnetic bead. The respiration rate was calculated by a linear regression when a monotonic decline in oxygen was observed. For nonlinear declines, the respiration rate was calculated after 1 h of incubation using the first derivative function of a second-order polynomial. Respiration curves were defined as nonlinear when the fitted *R*^2^ value was 0.02 higher than when fitting a linear curve. Using this criterion as a guideline, ~ 67% of measurements were nonlinear, leading to an underestimation of the respiration rates if using a linear model (mean − 70%). Cell-specific bacterioplankton respiration (*R*_sb_) was calculated by dividing the bacterial respiration rate by bacterial cell abundance determined directly after 1.2-μm filtration. The variation coefficient between sample bottles from the same depth was estimated to be ± 9.5% based on individually sampled measurements from the same estuary (*n* = 8, data not shown).

### Bacterial Abundance

Bacterial abundance was estimated by direct epifluorescence microscopy according to Hobbie et al. [30]. Samples were collected to determine the total bacterial abundance prior to filtration, after filtration, and after 12 to 24 h of respiration measurements. Water was collected in 50-cm^3^ Falcon tubes rinsed with sample water and was preserved with 37% formaldehyde (2% final concentration); 5-cm^3^ sample aliquots were filtered onto 0.2-μm black-stained polycarbonate filters (25 mm, DHI) and stained with Acridine orange (10 mmol dm^−3^, Sigma-Aldrich) which enables proper whole-cell labeling for accurate cell volume calculation according to Blackburn et al. [31]. Cell abundance and cell volume were determined by epifluorescence microscopy (Axiovert 100, Zeiss GmBH Germany) using image analysis procedures [31]. The coefficient of variation between sample bottles was estimated to be ± 4.2%, based on separate samples from the same estuary (*n* = 33, monitoring data). Cell volume was converted to cell carbon density according to the functions described by Norland [32] and Simon and Azam [33] for calculating bacterial biomass production in carbon units. One bacterial abundance sample was lost during fixation, reducing the number of samples for calculating *μ* in April (from *N* = 20 to *N* = 19).

### Bacterial Growth

Bacterial community biomass production (*P*_b_) was determined in triplicate subsamples directly after 1.2-μm filtration using the thymidine incorporation method at an in situ temperature in the same water baths as described for the respiration measurements [34]. Thymidine incorporation was selected because the rate of cell division is relevant for measuring the bacterial specific growth rate (*μ*), and season-specific conversion factors for cell production were available. In addition, using the thymidine method standardized the procedure for biomass calculation between bacterial biomass and the bacterial biomass production reducing uncertainty. The choice of a trace compound should, however, not be critical when using empirical conversion factors, as thymidine and leucine incorporation then provide comparable estimates of bacterioplankton production [35]. A 1-cm^3^ water sample was added to an Eppendorf tube, and 100 mm^3^ ice-cold TCA (50%) was added to the controls to stop growth. ^3^H-thymidine (2 mm^3^, 86 mCi mmol^−1^, 20 nM final concentration; GE Healthcare, Buckinghamshire, UK) was added to the samples and controls, which were then incubated for 1 h. Uptake of ^3^H-thymidine was converted to cell production using a season-specific thymidine conversion factor (TCF) of 1.2 × 10^18^ (April, ±SE 0.66 × 10^18^, *n* = 3) or 1.7 × 10^18^ (August, ±SE 0.21 × 10^18^, *n* = 2) cells [mol thymidine]^−1^, where the August TCF was 46% higher than the April TCF. The same conversion factor was assumed applicable for all samples during each transect. The estimated TCFs were close to reported values from the same environment [11]. The seasonal TCFs were determined by simultaneous measurement of thymidine uptake and bacterial abundance in duplicate batch cultures with reduced predator abundance by dilution in 0.2-μm filtered sea water (1:3, unpublished data). For the numerical analysis, the modified derivative analysis method was applied according to Kirchman and Ducklow [36]. For conversion to carbon units, the cell carbon density determined above for bacterial biomass, based on the cell volume calculation for each sample, was multiplied by cell production. The same method was used for the field estimates of in situ bacterial biomass production in unfiltered samples (i.e., monitoring data). The bacterial biomass production estimates had a median coefficient of variation between sample bottles from the same depth of ± 6.4%, based on separate samples from the same estuary (*n* = 60, monitoring data). The bacterial specific growth rate (*μ*) within 1 h of the 1.2-μm filtration was calculated by dividing bacterial production with bacterial abundance, both in cell units. The *μ* in filtered samples in this study was compared with *μ* in unfiltered samples derived from monitoring data (see below) from the same month and depth layer (*n* = 4). Medians of specific growth rate were compared using an independent-samples *k*-sample median test with SPSS™ (v.23) software.

### Bacterial Growth Efficiency

BGE was determined by the function

4 $$ \mathrm{BGE}=\frac{P_{\mathrm{b}}}{P_{\mathrm{b}}+{R}_{\mathrm{b}}} $$

where *P*_b_ is the measured bacterial community biomass production in carbon units, and *R*_b_ is the measured bacterial community respiration within an hour of filtration of the sample. A respiration quotient (RQ) of 0.9 was applied to convert O_2_ consumption to carbon units [37].

### Nutrients and Dissolved Organic Carbon

Fifty cubic centimeters of the 1.2-μm filtrate at each station and depth was collected and stored in Falcon tubes pre-rinsed with Milli-Q water. The samples were filtered through 0.2-μm sterile filters, filtrate oxidized with potassium peroxodisulfate, autoclaved, and stored at room temperature. Analysis was done within a week using an accredited standard operating procedure at Umeå Marine Sciences Centre to measure total dissolved phosphorus (TDP) and total dissolved nitrogen (TDN). The method described by Grasshoff et al. [38] was used, including a four-channel autoanalyzer (QuAAtro Marine, Bran & Luebbe®, Sweden). The median variation coefficient between samples from the same depth was estimated to be ± 2.1% for TDP and ± 8.2% for TDN based on samples from the same estuary.

The concentration of DOC was determined by transferring 60 cm^3^ subsamples from the 1.2-μm filtrate to rinsed hydrophilic surface-coated polystyrene bottles (Nunc®, Thermo Fisher Scientific) according to accredited and calibrated standard operating procedures at Umeå Marine Sciences Centre. Duplicate subsamples with a volume of 30 cm^3^ were filter sterilized (0.2 μm, 25 mm *Ø*, Supor filters, Pall Corporation), acidified with HCl to an approximate pH of 4, and stored in the dark at 8 °C for less than a week. The DOC was determined using a high-temperature catalytic oxidation instrument with nondispersive infrared (NDIR) detection and a TOC-L instrument (Schimadzu Corporation, Kyoto, Japan) [39, 40]. The median variation coefficient between samples collected from the same depth was estimated to be ± 2.2% for the DOC based on samples from the same estuary.

### Correlation Analysis

The correlation analysis was conducted using IBM SPSS™ (version 22). The data were generally not normally distributed (Shapiro-Wilks test) within months or across transects. Transformations using the inverse and the natural logarithm failed to transform all independent variables to fit a normal distribution. Therefore, a nonparametric Kendall’s tau two-tailed test with Bonferroni correction for multiple comparisons was used to determine the correlation coefficient *r*_*τ*_. The data were analyzed per depth layer (April *n* = 10, August *n* = 11) and for the depth layers combined (April *n* = 20, August *n* = 22) for both productivity conditions. One bacterial sample from April was lost. Due to the data distribution, parametric tests (including multiple factors) were not applicable.

## Results

### Environmental Conditions and Range of Variables

The average temperature was 3.1 °C in April and 13.9 °C in August, showing a 3 °C difference within the transect in April and a 10 °C difference in August (Table S1, Fig. 2). The bacterial biomass production in situ co-varied with the temperature, where low- and high-productivity conditions for bacteria were observed in April and August, respectively (Fig. 2). Salinity was higher in April (4.18 g kg^−1^) than in August (2.97 g kg^−1^), showing a similar range from 0 to 5 (± 0.5) g kg^−1^ over the whole transect in both productivity conditions.

TDP was approximately 40% higher in April than in August and showed a 7.7-fold range in April and a 3.8-fold range in August (Table S1). The corresponding ranges for TDN were 2.6-fold in April and a 3.8-fold range in August, while median values were similar between productivity conditions. DOC was 61 μmol dm^−3^ higher in August than in April, showing a similar 3-fold range within transects. The corresponding range for the C:P ratio was 9-fold in both April and August, demonstrating a 47% higher median in August. The observed nutrient ratios for both carbon and nitrogen versus phosphorus were high compared to the stoichiometric molar ratios expected for bacteria (45 C:10 N:1 P, [41]) and phytoplankton (106 C:16 N:1 P, Redfield ratio, Table S1), respectively.

The bacterial specific growth rates in the 1.2-μm filtered samples varied 4-fold in April and 10-fold in August. Specific bacterial respiration (*R*_sb_) showed a 24-fold range in the river-to-offshore transect in April and a 6.3-fold range in August, with similar median values between the productivity conditions (Table S1). The median value of BGE for both depth layers was approximately three times lower in April (2%) compared to the value in August (7%, Mann-Whitney *U* test, *p* < 0.01, *n* = 20). All BGE values were below 18%, and the minimum values approached 0 at both productivity conditions.

The bacterial specific growth rate (*μ*) of free-living bacteria in the 1.2-μm filtrate was approximately 4 times lower in April than the growth rates measured in unfiltered samples during the same month (Table 1). For August, the values were 3 times lower in the filtered samples. The effect of filtration was statistically significant for both months at both depth levels (*k*-sample median test, *p* < 0.05), with the exception of April below the pycnocline, which was slightly above the conventional significance level for both depth layers (*k*-sample median test, *p* = 0.07). The latter was related to a higher standard deviation for the August samples for both the filtered transect samples and unfiltered monitoring data. The filtered samples in August still covered 75% of the range of *μ* in unfiltered samples over the full year and water column (monitoring data, Fig. 2, lower panel). Minimum values in filtered samples were in general clearly lower than in the unfiltered samples (Table S1).

**Table 1:** Median values of bacterial specific growth rate (*μ*) in the 1.2-μm filtrate compared with field (monitoring) data measured in the same month in unfiltered water samples from the Öre Estuary in 2015. Maximum values (Max.), minimum values (Min.), standard deviation (SD), and number of samples (*n*) are shown

Month	Treatment	Surface water *μ* (day^−1^)					Deep water *μ* (day^−1^)				
		Median	Max.	Min.	SD	*n*	Median	Max.	Min.	SD	*n*
April	< 1.2 μm	0.032	0.043	0.013	0.010	11	0.018	0.031	0.007	0.007	9
	Whole water	0.13	0.16	0.12	0.023	4	0.10	0.11	0.075	0.017	4
	Whole w./ < 1.2 μm	2.6					2.9				
August	< 1.2 μm	0.047	0.20	0.013	0.069	12	0.015	0.17	0.002	0.052	10
	Whole water	0.26	0.28	0.24	0.015	4	0.10	0.14	0.072	0.035	4
	Whole w./ < 1.2 μm	4.8					7.5				

### Relationships Between Bacterial Specific Respiration and *μ*

**Fig. 3:**
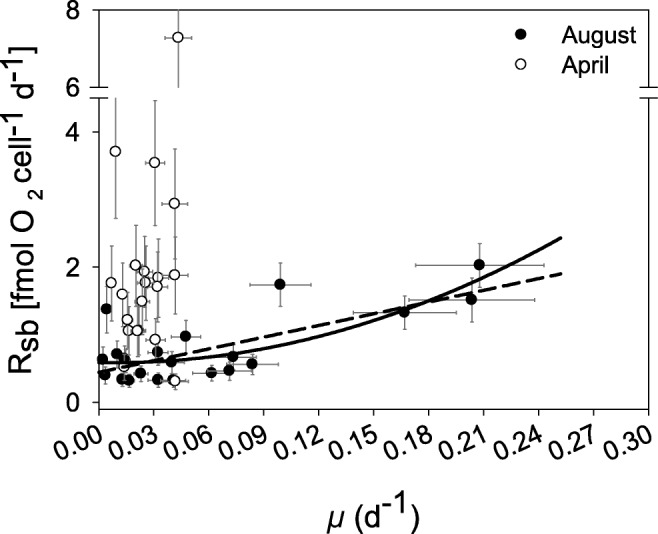
Bacterial cell-specific respiration rate (*R*_sb_) plotted against bacterial cell-specific growth rate (*μ*) for both months. The dashed line shows the significant model II regression for August (Eq. 2). The solid line shows the fit for the quadratic model (Eq. 5). Error bars show 2 × SD calculated from replicate sample bottles in the monitoring data set

Only the August data showed a significant linear relationship between *R*_sb_ and *μ* (*R*^2^ = 0.45, model II regression, Fig. 3). The *y*-intercept corresponded to a *R*_m_ of 0.32 (± 95% CI 0.18) fmol O_2_ day^−1^ cell^−1^ and a linear slope coefficient (*b*_l_) of 8.0 ± 95% CI 2.96. A nonlinear regression with a quadratic polynomial model resulted in a better coefficient of determination (*R*^2^ = 0.57):

5 $$ {R}_{\mathrm{sb}}={R}_{\mathrm{m}}+{b}_{\mathrm{n}}\times {\mu}^2 $$

The *y*-intercept coefficient was statistically significant (0.58 fmol O_2_ day^−1^ cell^−1^, 95% trimmed range 0.44–0.78), as was the nonlinear coefficient *b*_n_ (28.7 fmol O_2_ day cell^−1^, 95% trimmed range 18.6–41.3). Trimmed range is the uncertainty measure of nonlinear analysis due to bootstrapping methodology. Neither April alone nor both months combined showed a statistically significant relationship between *R*_sb_ and *μ*. However, the April data clearly demonstrated higher respiration rates in individual samples than the data from August (Fig. 3, cf. 95% CI). The average specific bacterial respiration in April was also significantly different from 0, at 1.75 fmol O_2_ day^−1^ cell^−1^ (± 95% CI 0.45), which was approximately 3 times higher than the *R*_m_ for the August data and twice the mean for *R*_sb_ in August (0.81 fmol O_2_ day^−1^ cell^−1^ ± 95% CI 0.22).

Most values for *μ* observed in April were in the lower range of the August values, as expected, but this difference was not statistically significant (Mann-Whitney *U* test, *p* > 0.05, *N* = 19, one lost sample). Thus, despite similar *μ* values for samples from April and August, the bacterial cell-specific respiration was, on average, higher in April (Mann-Whitney *U* test, *p* < 0.01, *N* = 19). Heterotrophic productivity was higher in August, as verified by the significantly higher bacterial biomass production both in filtered samples (Mann-Whitney *U* test, *p* < 0.01, *N* = 19, Fig. 2 upper panel, Table S1) and in unfiltered samples from the field (*t* test, *p* < 0.05).

**Fig. 4:**
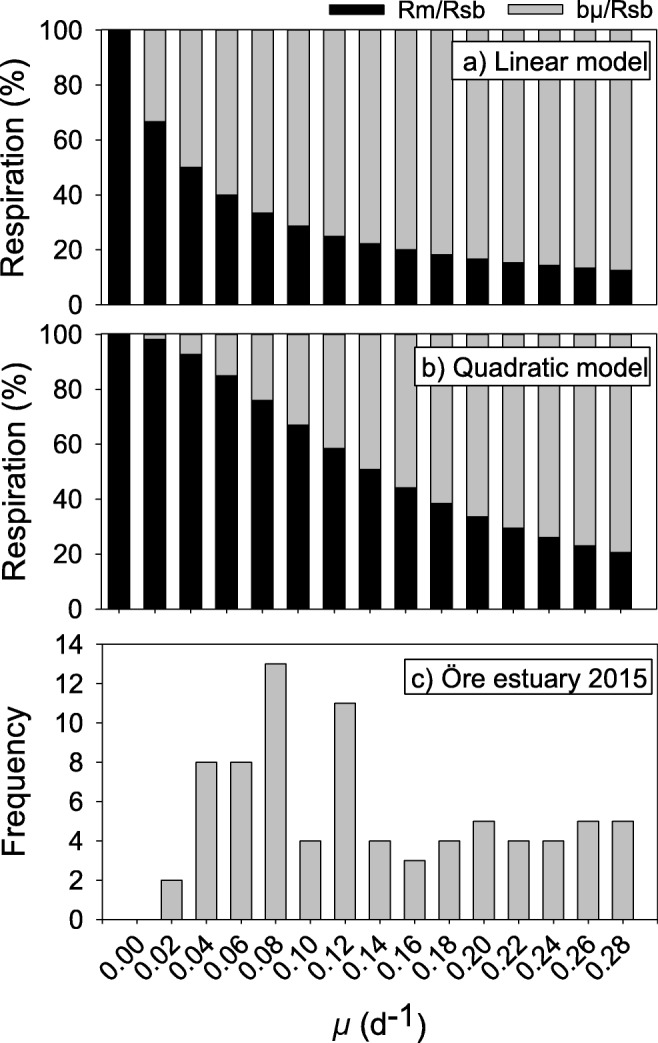
**a** Contribution of maintenance respiration (*R*_m_/*R*_sb_) as a function of bacterial growth rate (*μ*) over the observed range in filtered samples in the August transect. The *R*_m_/*R*_sb_ ratio was calculated from the linear model II regression function obtained in August (Eq. 2). **b** Same as **a** but based on the quadratic model obtained in August (Eq. 5). **c** Histogram showing the number of measurements for given bacterial cell-specific growth rate (*μ*) intervals in natural unfiltered samples over a whole year (2015) and water column of the Öre Estuary (monitoring data)

To assess the potential contribution of maintenance respiration at different specific growth rates, the relative contribution of *R*_m_ versus that of *R*_sb_ was calculated for the set of *μ* values observed in the monitoring data of the Öre Estuary 2015. Specific growth rates from the monitoring data spanning January to December of 2015 and the whole water column were aggregated in intervals of 0.02 day^−1^ (i.e., a histogram, Fig. 4). Calculations of maintenance respiration (*R*_m_) according to the modified linear Pirt model (Eq. 2) showed that *R*_m_ amounted to over 40% of *R*_sb_ at *μ* less than 0.06 day^−1^, declining exponentially with increasing *μ* (Fig. 4a). Using the coefficients from the empirically derived quadratic model, *R*_m_ amounted to 85% of *R*_sb_ at the same *μ*, showing a sigmoidal increase as *μ* declined (Fig. 4b). It was assumed in this calculation that growth-related maintenance respiration remained unchanged at higher growth rates (rather than decreasing), suggesting a possible overestimate of *R*_m_ at a higher *μ*.

To prove the concept regarding the contribution of *R*_m_ on an annual scale, the calculated *R*_m_/*R*_sb_ was integrated over the year 2015 and over the whole water column for the Öre Estuary (Eq. 3). The obtained histogram of bacterial specific growth rates (Fig. 4c) showed that the range of *μ* observed in August covered the majority (75%) of the *μ* range measured during 2015. Thus, bacterial specific growth rates measured in samples collected during a week in August, covering a land-sea transect and different depth layers, matched the range found during a full year for unfiltered samples from this subarctic estuary. By using the relationship of *R*_m_*/R*_sb_ values and *μ* in Fig. 4a (linear model), we calculated a weighted annual average contribution of *R*_m_ to *R*_sb_ (Eq. 3) of 24% for the Öre Estuary in 2015. Using the coefficients from the nonlinear model (Eq. 5), the influence of maintenance respiration on an annual level increased to 58%.

### Correlations between Respiration, Nutrients, and Temperature

**Fig. 5:**
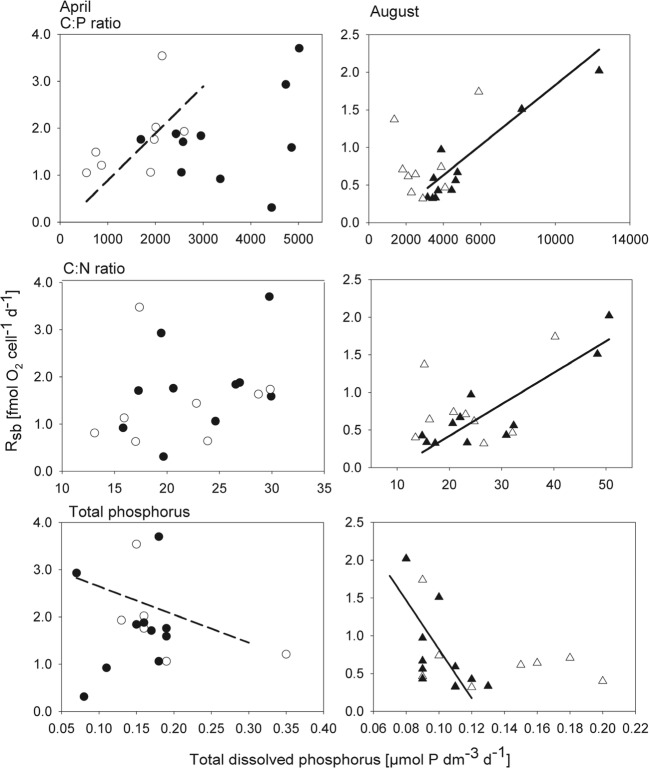
Presentation of relationships between *R*_sb_ and the measured nutrient variables. Relationships with a significant (*p* < 0.05) Kendall’s tau correlation coefficient of *r*_*τ*_ > 0.5 are indicated by a line. A solid line and filled symbols show surface water, while deep water is shown by a dashed line and open symbols. Lines are derived by model II regressions. No significant correlations were found for total nitrogen

Based on the linear influence of nutrients, and especially of phosphorus, on bacterial respiration postulated by the Pirt model, we examined linear correlations with nutrient concentrations and their ratios by nonparametric tests given the obtained data distributions. To distinguish between a situation rich in nutrients, relevant to the Pirt model, and poor conditions, the April and August data were evaluated separately. The strongest correlations with respiration in April were related to TDP (negative correlation, *r*_*τ*_ = − 0.51, *p* = 0.04, Fig. 5, Table 2) and the C:P ratio (positive correlation, *r*_*τ*_ = 0.69, *p* < 0.01, Table 2) in the deep layer. A positive correlation between *R*_sb_ and the C:N ratio, with a comparably low *r*_*τ*_ of 0.43 (*p* < 0.05), was found when depths were aggregated. In August, similar relationships to those in April were found for surface waters, with the strongest correlation between *R*_sb_ and the C:P ratio (*r*_*τ*_ = 0.67, *p* < 0.01). A somewhat weaker positive relationship was found between *R*_sb_ and the C:N ratio in the surface layer (August), along with a negative relationship relative to TDP (*r*_*τ*_ = 0.59, *p* = 0.01). No significant relationship between *R*_sb_ and temperature could be found on the investigated scale.

**Table 2:** Kendall’s tau *b* correlation coefficient (*r*_*τ*_) between bacterial specific respiration (*R*_sb_) and explanatory factors for both month and surface and deep water. Data with *p* values < 0.05 are shown in italics

Factor	*R*_sb_ April		*R*_sb_ August	
	Surface water	Deep water	Surface water	Deep water
Temperature	0.34	0.03	− 0.04	− 0.02
TDP	− 0.07	*− 0.51*	*− 0.59*	− 0.15
TDN	− 0.47	− 0.11	− 0.06	0.13
DOC	− 0.07	0.51	0.24	0.38
C:P ratio	− 0.07	*0.69*	*0.67*	0.13
C:N ratio	0.38	*0.56*	*0.53*	0.06
*μ*	0.38	0.16	*0.56*	0.09

## Discussion

Respiration is typically assumed to be directly related to growth and biomass synthesis. However, the level of maintenance respiration has potential importance for the level and control of the total ecosystem respiration and, thereby, may contribute to understanding the cause of the development of hypoxia, as well as the emission of CO_2_.

Our first question addressed was if the concept of bacterial maintenance respiration, as represented by the Pirt model, is of ecological significance in an aquatic bacterial community? To test this question and estimate the level of bacterial maintenance respiration, we applied the Pirt model on a data set covering a range of bacterial growth rates corresponding to the annual range in a subarctic estuary. Bacterial specific respiration (*R*_sb_) was demonstrated to be a linear function of the bacterial specific growth rate (*μ*) during high-productivity conditions, corroborating the validity of the Pirt model in the natural environment. The Pirt model was developed assuming energy-rich conditions and should therefore be valid for the high-productivity conditions in August, coinciding with the annual peak in maximum water temperature and well-developed phytoplankton and zooplankton communities (Fig. 2) [26]. Under these conditions, high abundances of protozooplankton and macrozooplankon provide a rich source of energy for bacteria via the exudation of organic substances and nutrients from vacuoles, inefficient feeding, and feces [42]. At lower productivity in early spring (April data), however, no significant relationship between *R*_sb_ and *μ* could be demonstrated. Given the limits of the Pirt model, developed during high-energy conditions, more knowledge of bacterial respiration under starvation conditions in natural environments is required.

We also demonstrated that a quadratic nonlinear model better described the observed relationship between *R*_sb_ and *μ* under high-productivity conditions (Eq. 5, R^2^ = 0.57, Fig. 3). The pattern for the August observations, when *μ* approached 0, clearly showed that bacterial respiration remained unchanged from undetectable rates up to a *μ* of 0.09 day^−1^. We therefore adopted the quadratic model as more reliable.

The *y*-intercept of the nonlinear model provides the first estimate, to our knowledge, of bacterial maintenance respiration in the field, with a value of 0.58 fmol O_2_ day^−1^ cell^−1^. This was 1.8 times higher than when estimated by the linear Pirt model. The trimmed 95% range suggests the true value lies in between − 24% and + 34% of the estimate. The aggregation of observations at the lower end of the curve and the good fit to the quadratic model contribute to the confidence in the *R*_m_ estimate. Fewer data in the mid-range of the curve leave some uncertainty as to the validity of the nonlinear model at a higher *μ*. The maintenance respiration estimate reported by Cajal-Medrano and Maske [13], using the Pirt model on literature values, was three times higher than our value. Recalculating our maintenance respiration rate by measured cell density (1.5 fmol C cell^−1^, RQ = 1) gives a value of 0.39 day^−1^. Their estimate would therefore indicate an even higher influence of maintenance respiration. However, we question their estimate as respiration was calculated from *μ* and growth yield, leading to autocorrelation in their data set.

Under low-productivity conditions (i.e., April), bacterial respiration per cell unexpectedly exceeded respiration in August by approximately a factor of 3, and no relationship to the bacterial specific growth rate could be demonstrated (Fig. 3). The observed lack of a relationship between *μ* and *R*_sb_ in the 1.2-μm filtrates unambiguously demonstrates that bacterial respiration can be significant and uncoupled from contemporary biomass growth requirements. Consequently, the Pirt model could not explain the observations during low-productivity conditions. We suggest that these specific respiration rates are primarily due to maintenance respiration. Hence, we conclude that *R*_m_ was responsible for nearly all of the *R*_sb_ under low-productivity conditions in April, reducing BGE under low-productivity conditions.

It remains unclear what bacterial communities are spending their energy on during periods of low productivity in the natural environment, despite extensive knowledge about bacterial adaptations at low-energy conditions in laboratory cultures [43]. We can only speculate that elevated maintenance energy requirements drive activities to gain competitive advantages such as the production of external enzymes, formation of storage material, or flagellar motion, as previously reported [9, 43, 44]. Bacteria in continuous cultures also show energy-spilling reactions, suggested to be related to removal of toxic end products or priming of metabolites for future anabolism, possibly contributing to maintenance respiration [44].

Our second question addressed how important maintenance respiration is when compared to total bacterial respiration. The contribution of *R*_m_ to *R*_sb_ was calculated from the relationships above (Eqs. 2 and 5) and plotted versus *μ* (Fig. 4). Both models showed that bacterial maintenance respiration constituted a significant share of total respiration on the order of tens of percent. At the median *μ* (0.14 day^−1^) of the range analyzed, *R*_m_/*R*_sb_ was 20% when using the linear Pirt model. For the empirically derived model, this was even more pronounced, with a *R*_m_/*R*_sb_ quotient of approximately 50% at the same *μ*. This corresponded to the 20–80% observed for *Klebsiella aerogenes* in continuous culture, depending on whether glucose or phosphate was limiting, used in the development of the Pirt model [12].

To investigate the contribution from *R*_m_ on an annual scale, we calculated a minimum value using Eq. 3 and the range of *μ* measured in the active environmental monitoring program (cf. Fig. 2). Assuming that *μ* was the major predictor of *R*_m_ and *R*_sb_, as found above for the high-productivity conditions in August (i.e., Eq. 2), *R*_m_ accounted for 24% of the annual bacterial respiration, and when using the empirically derived coefficients from the quadratic model (Eq. 5), this value increased to 58%. We conclude from this that bacterial maintenance respiration can make up a significant part of annual bacterial respiration in this subarctic estuary.

The quadratic model more accurately represented *R*_m_ when *μ* approached zero growth. We may still have overestimated the influence of *R*_m_ since the constant maintenance cost (*m*_1_) was not discernible from the growth-dependent maintenance cost (*m′*, cf. Eq. 1). In contrary, the higher share of *R*_m_ under the low-productivity conditions (April data) means a risk to have underestimated *R*_m_ influence on an annual basis, assuming this *R*_m_ is valid for the unproductive half of the year. Hence, with a conservative assumption that these uncertainties cancel out, maintenance respiration is likely to account for at least half of the annual bacterial respiration in this subarctic estuary.

No clear relationship to *μ* could be demonstrated in April. Instead, *R*_sb_ varied substantially, showing unexpectedly high cell-specific respiration rates compared with the observed low *μ*. This inconsistency leads to the third question addressed in the “Introduction,” to what extent does nutrient stoichiometry explain the level of maintenance respiration in the field. Based on the conclusions of Neijssel and Tempest [12], we hypothesized that *R*_sb_ could be stimulated by an increase in the C:P ratio, and for the Öre Estuary, phosphorus is the limiting nutrient [24, 45, 46]. Nutrient ratios also indicated marked phosphorus limitation (Table S1). Significant relationships between *R*_sb_ and both TDP (negative) and the C:P ratio (positive, squared *r*_*τ*_ = 36%) in deep water implied a higher cellular respiration demand at phosphorus shortage as postulated by the Pirt model. For the surface layer, no consistent relationship could be demonstrated in April between *R*_sb_ and nutrients or their ratios. In August, the C:P ratio could explain as much as 49% of the variation in *R*_sb_ in the surface water, and a weaker correlation with TDP was also observed, but no correlation was found in the deep layer (Table 2, Fig. 5). Thus, our results could only partly support a role of phosphorus in imposing high maintenance respiration rates as found in the laboratory experiments of Neijssel and Tempest [12], and factors other than TDN, TDP, DOC, and their ratios should explain more than half of the variation in *R*_sb_ during periods of low productivity.

Similar effects of phosphorus and carbon on bacterial respiration in natural environments have been observed in other environments. Smith and Prairie [19] reported a marked increase in *R*_sb_ as the phosphorus concentration decreased in North American lakes, in line with our findings. Supporting our interpretation, Smith and Kemp [20] also found that increased carbon levels stimulated respiration in the lower part of the Chesapeake Bay, while a decrease in phosphorus resulted in an increase in bacterial specific respiration. In other parts of the Chesapeake Bay, however, no effect of either carbon or phosphorus addition could be demonstrated. Given that current literature and our results show a variable response of *R*_sb_ in relation to nutrient stoichiometry in the field, we cannot make general conclusions based on the present observations.

Temperature was not significantly correlated to *R*_sb_ in our study, in contrary to other reports [17]. We conclude that (1) temperature was of less importance as significant in the control of bacterial respiration by two discrete productivity levels and (2) the lack of a relationship can primarily be explained by the limited span of temperatures present in April (Δ*t* = 3 °C). The range of both nutrients (3- to 9-fold) and bacterial variables (6- to 24-fold), however, was significant. The spatiotemporal scale of this study, our short-term measurement directly after filtration, and the use of natural nutrient levels, may explain differences in results compared to studies using incubation over days in small sample volumes with nutrient amendments (e.g., [22]).

### Experimental Design and Validity of Data

Our experimental design to study filtered samples was imposed by the need to specifically measure bacterial respiration, and it limits the generalizability of our results to free-living bacteria. By using estimates within 1 h after filtration, the effects of enclosure during incubation time or experimentally induced activity of natural viruses should be minor. The observed low *μ* following filtration (Table 1), however, indicated that the prefiltration resulted in degraded growth conditions in comparison to bacterioplankton growth in unfiltered samples (compared with monitoring data, Fig. 2). We probably deprived bacteria of continuous supply of nutrients from protozoan and zooplankton egestion and nutrient-rich particles thereby lowering *μ*. That particle-attached bacteria show higher activity and different taxonomy than free-living has been shown in other estuaries [47, 48]. The span of specific growth rates remaining in the August study was still sufficient to cover the majority (75%) of the *μ* range observed during a year in the estuary (Figs. 2, 3, and 4c). Given that *μ* constitutes a major predictor for bacterial respiration by both laboratory experiments [12] and field studies [5], our results are relevant for the natural environment. In April, a coverage of the natural range of *μ* was not met and we cannot exclude that a starvation response (i.e., stress), imposed by prefiltration, influenced the high respiration rates observed. The observations in April still show that bacterial respiration can be high at low growth rates.

The level of BGE observed suggests that the results from our filtered samples are relevant for the natural environment and applicability to natural samples. The average BGE value of 0.09 (August) was close to that found by Wikner et al. [49] (i.e., median 0.11), who used a different method to calculate BGE in the same water body (i.e., bacterial carbon yield and DOC net change). The observed level of BGE also results in a carbon budget for the estuary better in line with plankton respiration recently reported [50, 51]. Both these comparisons suggest that bacterial growth and respiration measurements in the filtered samples were realistic and match global models for BGE versus bacterial biomass production [5, 10].

### Potential Impact of Maintenance Respiration

**Fig. 6:**
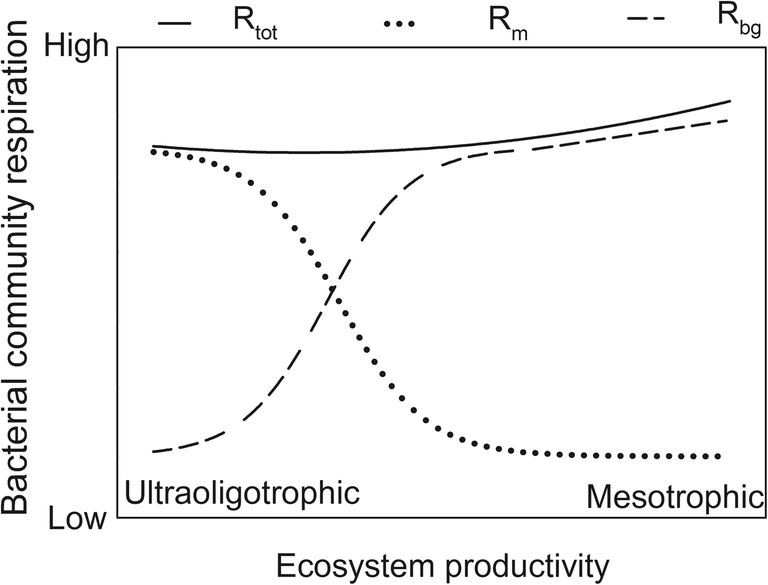
Conceptual model of the proposed effect of ecosystem productivity (e.g., changes in the limiting nutrient) on total bacterial respiration (*R*_sb_), biomass growth-related respiration (*R*_bg_), and maintenance respiration (*R*_m_) in an aquatic environment. The hypothetical ecosystem productivity interval used represents ultraoligotrophic to mesotrophic level

The strong influence of maintenance respiration found has an important consequence at the ecosystem level. This is because a moderate increase in ecosystem productivity will not result in increased oxygen consumption. Instead, a shift from maintenance- to growth-based respiration occurs, leaving total oxygen consumption unchanged (Fig. 6). An increase in total community respiration thus requires that either bacterial specific growth rate becomes sufficiently high, bacterial abundance increases, or a combination of both occurs. That is a scenario expected in eutrophic environments. As a result, considering the opposite trajectory, nutrient reductions may not necessarily lead to reduced oxygen consumption and improved oxygenation of hypoxic environments. Instead, in low-productivity environments, a shift from respiration related to biomass synthesis to maintenance respiration may occur, with a minor influence on total respiration.

## Electronic Supplementary Material

Table S1 (DOCX 18 kb)
