# Supplementary material for: Importance of Bacterial Maintenance Respiration in a Subarctic Estuary: a Proof of Concept from the Field
Source: Microb Ecol. 2018 Aug 22;77(3):574–86. doi: 10.1007/s00248-018-1244-7 (PMC6469616; doi:10.1007/s00248-018-1244-7)
Supplement: Supplementary file 1 — (DOCX 18 kb) [file 248_2018_1244_MOESM1_ESM.docx]

**Legends for supplementary information**Table S1. Descriptive data of measured variables in 1.2 µm filtered water samples. Standard deviation (SD) and number of measurements (n), after outliers were removed, are shown.

|  | Mean | | Median | | Maximum | | Minimum | | SD | | n | |
| --- | --- | --- | --- | --- | --- | --- | --- | --- | --- | --- | --- | --- |
|  | April | August | April | August | April | August | April | August | April | August | April | August |
| Temperature (°C) | 3.11 | 13.9 | 3.00 | 16.0 | 4.00 | 17.0 | 2.50 | 7.00 | 0.39 | 3.42 | 20 | 22 |
| Salinity (g kg^-1^) | 4.18 | 2.97 | 4.80 | 2.93 | 5.40 | 4.7 | 0.02 | 0.00 | 1.67 | 1.37 | 20 | 22 |
| TDP (µmol dm^-3^) | 0.20 | 0.12 | 0.17 | 0.11 | 0.54 | 0.30 | 0.07 | 0.08 | 0.12 | 0.05 | 20 | 22 |
| TDN (µmol dm^-3^) | 18.9 | 21.2 | 17.8 | 18.2 | 30.8 | 47.8 | 12.0 | 12.7 | 5.26 | 8.94 | 20 | 22 |
| DOC (µmol dm^-3^) | 409 | 470 | 342 | 397 | 923 | 1013 | 297 | 320 | 177 | 202 | 20 | 22 |
| DOC:TDP ratio | 2585 | 4392 | 2489 | 3647 | 5017 | 12351 | 554 | 1368 | 1383 | 2834 | 20 | 22 |
| DOC:TDN ratio | 21.8 | 24.9 | 20.9 | 21.9 | 32.8 | 50.6 | 12.7 | 9.3 | 5.9 | 12.3 | 20 | 22 |
| TDN:TDP ratio | 117 | 175 | 101 | 169 | 243 | 252 | 34.3 | 91.0 | 59.2 | 47.9 | 20 | 22 |
| *R_sb_* (fmol O_2_ cell^-1^ d^-1^) | 1.98 | 0.78 | 1.73 | 0.62 | 7.27 | 2.02 | 0.31 | 0.32 | 1.52 | 0.50 | 20 | 22 |
| *µ* (d^-1^) | 0.03 | 0.06 | 0.02 | 0.04 | 0.04 | 0.21 | 0.01 | 0.01 | 0.01 | 0.06 | 19 | 22 |
| Bacterial respiration (µmol O_2_ dm^-3^ d^-1^) | 2.78 | 2.60 | 2.47 | 2.14 | 4.68 | 6.43 | 0.46 | 0.94 | 1.25 | 1.5 | 20 | 22 |
| Bacterial biomass prod.  (µmol C dm^-3^ d^-1^) | 0.06 | 0.28 | 0.05 | 0.17 | 0.10 | 1.03 | 0.02 | 0.01 | 0.03 | 0.30 | 20 | 22 |
| BGE | 0.03 | 0.09 | 0.02 | 0.07 | 0.17 | 0.17 | 0.01 | 0.01 | 0.04 | 0.06 | 20 | 22 |
| Chl *a* > 10 µm | 6.4 | 2.6 | 6.7 | 2.7 | 10.1 | 6.9 | 0.8 | 0.3 | 1.7 | 1.0 | 265 | 562 |
